# Short-Term Impact of a Comprehensive Smoke-Free Law Following a Partial Smoke-Free Law on PM_2.5_ Concentration Levels at Hospitality Venues on the Peripheries of College Campuses

**DOI:** 10.3390/ijerph121114034

**Published:** 2015-11-02

**Authors:** Sol Yu, Wonho Yang, Kiyoung Lee, Sungcheon Kim, Kwonchul Ha, Sungroul Kim

**Affiliations:** 1 Department of Environmental Health Sciences, Soonchunhyang University, Asan 336-745, Korea; E-Mail: solsol0914@gmail.com; 2 Department of Occupational Health, Catholic University of Daegu, Gyeongsan-si 712-702, Korea; E-Mail: whyang@cu.ac.kr; 3 Department of Environmental Health and Institute of Health and Environment, Graduate School of Public Health, Seoul National University, Seoul 151-742, Korea; E-Mail: cleanair@snu.ac.kr; 4 Department of Environmental Engineering, Kunsan National University, Seoul 110-810, Korea; E-Mail: ksc@kunsan.ac.kr; 5 Department of Biochemistry & Health Science, Changwon National University, Changwon 641-773, Korea; E-Mail: kcha@changwon.ac.kr

**Keywords:** secondhand smoke, college campus, hospitality venues, PM_2.5_

## Abstract

Tobacco-free policies on college campuses are spreading in Korea. However, the impact of the smoking ban legislation at venues on the boundary of a college campus is still limited. This study aimed to assess short-term changes in PM_2.5_ concentrations before and after the enactment of the smoking ban legislation to evaluate the impact. In this cross-sectional study, PM_2.5_ measurements (pre-ban: *n* = 99, post-ban: *n* = 64) were conducted in randomly selected billiards, pubs, and computer game rooms on the peripheries of college campuses in October 2013, prior to implementation of the smoking ban, and in May 2014, after the ban. The median (interquartile range, IQR) of the PM_2.5_ concentration for all venues was 31 μg/m^3^ (0–80 μg/m^3^) in the pre-ban period and 11 μg/m^3^ (0–36 μg/m^3^) in the post-ban period implying indoor PM_2.5_ concentration levels of the peripheries of college campuses likely decreased one year after implementation of the ban. However, the differences were not significant yet. The results support the introduction of more rigorous monitoring of SHS exposure levels toward the ultimate goal of encouraging a complete smoking ban in hospitality venues, including billiards, pubs, and computer game rooms located on the peripheries of college campuses.

## 1. Introduction

Exposure to secondhand smoke (SHS) is a worldwide public health problem. In 2002, the International Agency for Research on Cancer (IARC) concluded that “involuntary smokers are exposed to the same numerous carcinogens and toxic substances that are present in tobacco smoke produced by active smoking”. In 2003, under the auspices of the World Health Organization, the Framework Convention on Tobacco Control (WHO FCTC) was introduced as the first international treaty [1]. It was signed by 168 countries and is legally binding in 180 ratifying countries, including Korea.

According to the FCTC, in Korea, Article 8 of the Enforcement Rules of the National Health Promotion Act was passed in 2012 for a complete smoking ban at hospitality venues, including computer game rooms, pubs, and restaurants where SHS levels were relatively higher than in other places [2,3]. In Korea, the Ministry of Health and Welfare introduced a legislation that regulates smoking in those enclosed hospitality venues, *i.e.*, computer game rooms and pubs and restaurants with indoor floor areas of 100 m^2^ or larger in January 2014 [2]. In Korea, since billiard rooms are classified as sports facilities, rather than a public place, only those billiard venues which can be occupied by 1000 people, are targeted for a complete smoking ban although most billiards spaces have much smaller numbers of occupants. Thus, there is expectation of having smokers inside small billiard rooms and it is highly doubtful that the policy would work effectively. Therefore, initial evaluation of the policy introduction is very important to determine the impact of the policy and to provide future directions for regulatory goals, especially for different places, *i.e.*, computer game rooms, pubs and billiards, frequented by young adults.

In Korea, the number of computer game rooms, pubs, and restaurants are increasing throughout many campus towns, since more than half of 20–25 year-old Koreans are university or college students and various social parties or activities occur there [4]. In America, college students tend to define themselves as “social or occasional smokers”—those who smoke a few cigarettes per day—but who use tobacco in social parties or activities, mostly at hospitality venues and find it essential for socializing [5,6].

In Korea young adults are 3 to 5 times more likely to smoke, compared to people aged 65 years and above [7]. Also among young Koreans, social smoking is increasing and it has been reported that one of main reasons for an individual to relapse into smoking was social smoking due to the surroundings and the most likely place for the relapse to occur was a drinking-related site [8].

It is well known that the main target of tobacco industry marketing is youths. The tobacco industry marketers have strategies increasing in cigarette consumption by focusing on key transition periods when young adults adopt new behaviors—e.g., entering a new school, or the military—and, especially, by focusing on leisure and social activities [9,10]. Therefore, in Korea, with consideration of the relatively large number of college students in their 20s understanding the degree of observance of the smoking ban legislation at venues on peripheries of a college campus can help public health practitioners develop better tobacco control programs and physicians to encourage nonsmoking among young adult regular or social smokers.

Tobacco-free policies on college campuses have already been enforced in Korea. However, the impact of the recent smoking ban legislation at hospitality venues on boundary of college campuses is still limited. This study, therefore, aimed to assess short-term changes in PM_2.5_ concentrations before and after the enactment (January 2014) of the smoking ban legislation with a 1-year interval using data obtained from hospitality venues in peripheries of college campuses in Korea.

## 2. Methods

### 2.1. Study Sites

Indoor PM2.5 measurements were conducted in billiard halls, pubs, and computer game rooms, randomly selected from three campus towns located in the megacities of Seoul, Daegu, and Asan in Korea, before (2013, *n* = 99) and after (2014, *n* = 66) implementation of the smoking ban legislation. We visited hospitality venues in each college campus periphery between October and December 2013 (before the implementation) and from May 2014 (after the implementation). The same venues from the pre-ban period were also used post-ban but 21 venues, accessed in 2013, had closed by 2014 and 12 venues were not available for inconspicuous measurement in 2014. The total number of smokers inside a venue, the indoor area measured using an ultrasonic distance meter (Zircon, Campbell, CA, USA) and the number of ventilators (simple air vents) in each venue were noted.

### 2.2. Sampling Method

We measured PM_2.5_ using a battery-operated real-time aerosol monitor (Sidepak AM510; TSI, Shoreview, MN, USA). We used 0.3 as a correction factor for measurements. Every afternoon prior to PM_2.5_ monitoring, we performed zero calibration and checked the flow rate (1.7 L/min) under standard operating procedures. Since most venues in campus towns are occupied by young adults from the late afternoon, we conducted our monitoring after 4 pm. The monitor was set to record PM_2.5_ concentrations every second, and then average them to provide mean values for every minute. Indoor PM_2.5_ concentration at each hospitality venue was measured for 30 minutes. Outdoor levels were measured for 5 minutes before and after measuring indoor PM2.5 levels at venues. Monitoring was conducted inconspicuously so as not to disturb users’ normal behaviors. Each monitor was placed on the central table in the sites. The inlet of the monitor was attached with a short length of Tygon tubing and left protruding outside. To minimize the effect of additional source contributions to our PM_2.5_ measurement results, we reported our PM_2.5_ results after subtracting the mean of the two field background PM_2.5_ concentrations from the values measured at the inside of each site. Inter/intra variations of PM_2.5_ monitors used for this study were tested before we used them for field studies.

### 2.3. Statistical Analysis

We compared the distribution of PM_2.5_ concentrations obtained randomly from each hospitality venue before (in 2013) and after (in 2014) the smoking ban. We used the Wilcoxon signed-rank test since the PM_2.5_ values were not normally distributed. We calculated a ratio of PM_2.5_ post-ban to pre-ban for each venue and then we provided a median value of the ratios for each category of venues. We then conducted separate analyses to examine the association between legislation and SHS exposure using multivariable regression models. Three different models were run. The first univariate model evaluated the association of PM_2.5_ concentration with existence of the smoking ban policy at each venue using the year as a binary variable (2014 = 1, 2013 = 0). The second model additionally included indoor area (m^2^) and the number of ventilators in different hospitality venues; billiard halls, pubs, and computer game rooms. The last model included the number of smokers, indoor area (m^2^), and the number of ventilators at each venue using multivariable models. We used a binary variable for area, *i.e.*, (1 = 100 m^2^ or greater, 0 = below 100 m^2^). The analyses were conducted using SAS version 9.3 (SAS Institute Inc., Cary, NC, USA).

## 3. Results

The median (interquartile range, IQR) of the number of smokers in all venues was 4 (1–9) and 0 (0–4) pre- and post-ban, respectively. The median (IQR) areas and numbers of ventilators were 204.5 m^2^ (107.5–360) and 10 (4–16) pre-ban, and 114.7 m^2^ (72.0–206.1) and 13 (7–18) post-ban, respectively. Other characteristics of the hospitality venues before and after implementation are summarized in Table 1.

**Table 1:** Summary of characteristics of sampling sites including area (m^2^), the number of smokers occupied and the number of ventilators installed inside.

		Total Number of Samples	Number of Samples < Background PM_2.5_ Level *N* (%)	Area (m^2^) Med (IQR)	Number of Smokers Occupied Med (IQR)	Number of Ventilator Med (IQR)
**Pre-ban (2013)**	**Overall**	99	23 (23.2)	204.5 (107.5–360)	4 (1–9)	10 (4–16)
	**Billiards**	30	5 (16.7)	232.0 (110.0–484.0)	3 (2–6)	8 (4–10)
	**Pubs**	30	10 (33.3)	132.5 (55.0–225.0)	2 (0–4)	5 (2–14)
	**Computer game rooms**	39	8 (20.5)	262.5 (156.0–368.0)	6 (4–13)	16 (9–26)
**Post-ban (2014)**	**Overall**	64	18 (28.1)	114.7 (72.0–206.1)	0 (0–4)	13 (7–18)
	**Billiards**	15	3 (20.0)	198.0 (117.6–286.0)	4 (3–6)	12 (9–16)
	**Pubs**	29	10 (34.5)	75.0 (54.0–112.8)	0	10 (3–15)
	**Computer game rooms**	20	5 (25.0)	141.2 (106.5–284.7)	1 (0–10)	19 (15–34)

**Figure 1:**
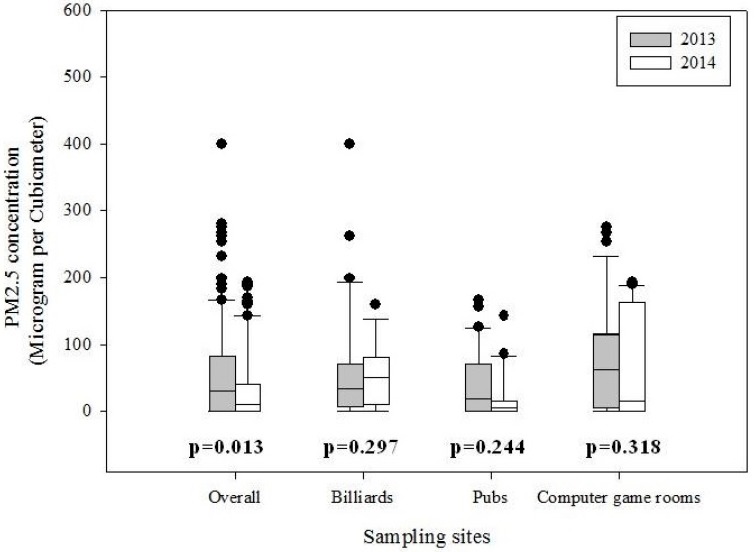
Comparison of distributions of PM_2.5_ concentrations measured between pre-ban (2013) and post-ban period (2014) (*p*-values from the Wilcoxon signed-rank test).

The median (interquartile range, IQR) of overall PM_2.5_ concentration was 31 μg/m^3^ (0–80) in 2013 and 11 μg/m^3^ (0–36) after a 1-year interval (*p* = 0.013). The median concentration for the two time points were 32.9 (6.1–68.4) μg/m^3^ and 49.8 (9.5–81.3) μg/m^3^ in billiards (*p* = 0.30); 18.8 (0–69.5) μg/m^3^ and 5.5 (0–15.3) μg/m^3^ in pubs (*p* = 0.24); and 61.7 (5.2–115.2) μg/m^3^ to 16.0 (1.8–161.8) μg/m^3^ in computer game rooms (*p* = 0.32), separately. The ratio of indoor PM_2.5_ concentrations for the post-ban to pre-ban periods was 1.51 for billiards, 0.29 for pubs and 0.26 for computer game rooms (Figure 1). Overall indoor PM_2.5_ concentrations were positively correlated with the number of smokers in 2014 (*r* = 0.62) as well as 2013 (*r* = 0.49) at most hospitality venues (Table 2).

**Table 2:** Spearman correlation coefficients between indoor PM_2.5_ concentration and other SHS-related variables, according to sampling period and venues.

	PM_2.5_ Concentration							
	Overall		Billiards		Pubs		Computer Game Rooms	
	Pre-ban	Post-ban	Pre-ban	Post-ban	Pre-ban	Post-ban	Pre-ban	Post-ban
**# of Smokers**	0.494	0.621 *	0.178	0.571 *	0.395 *	0.274	0.526 *	0.802 *
**Area (m^2^)**	0.117	0.175	−0.167	0.068	0.027	−0.086	0.300	0.047
**# of Ventilators**	0.174	0.296	0.378	0.229	0.005	0.159	0.137	0.365

* The values are statistically significant at the significance level of 0.05 with Spearman correlation analysis.

The short term impact of the smoking ban legislation in campus town venues was further evaluated by comparing PM_2.5_ concentrations after adjusting for the number of smokers, floor areas, and the number of ventilators. The univariate analysis (Model 1) showed that the PM_2.5_ concentrations for billiards, pubs, and computer game rooms in 2014 (post-ban) were 1.4, 0.2, and 0.5 times those in 2013 (pre-ban). In multivariable models for billiards or pubs, we could not find significant differences in PM_2.5_ concentrations between the two periods. However, for computer game rooms, we found that PM_2.5_ concentrations increased significantly from 3.3 to 239.6 times after controlling for other explanatory variables including the number of smokers, indoor area, and the number of ventilators (Table 3).

**Table 3:** Degree of change (95% CI) for indoor PM_2.5_ concentration level by potential determinants selected over pre- or post-ban period (values are coefficients from the regression models).

		Model 1	Model 2	Model 3
**Billiards**	**Existence of Policy (Ref: No)**			
	**Yes**	1.43 (0.16–13.02)	0.73 (0.07–7.83)	0.65 (0.06–7.59)
	**# of Smoker**	-		1.23 (0.81–1.86)
	**Area (m^2^)**	-	0.80 (0.03–19.80)	0.69 (0.03–18.34)
	**# of Ventilator**	-	1.14 (0.98–1.33)	1.11 (0.93–1.31)
**Pubs**	**Existence of Policy (Ref: No)**			
	**Yes**	0.24 (0.03–1.83)	0.38 (0.03–4.75)	0.28 (0.02–4.53)
	**# of Smoker**	-	-	0.94 (0.59–1.51)
	**Area (m^2^)**	-	2.60 (0.19–34.76)	3.60 (0.21–60.31)
	**# of Ventilator**	-	0.93 (0.81–1.08)	0.94 (0.81–1.11)
**Computer game rooms**	**Existence of Policy (Ref: No)**			
	**Yes**	0.52 (0.06–4.20)	0.37 (0.04–3.54)	27.86 (3.24–239.61)
	**# of Smoker**	-		1.33 (1.16–1.51)
	**Area (m^2^)**	-	1.53 (0.08–29.80)	13.40 (1.20–149.67)
	**# of Ventilator**	-	1.03 (0.94–1.14)	0.99 (0.92–1.07)

## 4. Discussion

Campus towns in urban areas in Korea feature a university and a diverse range of recreational venues including computer game rooms, pubs, clubs, and restaurants, considered the center of nightlife thus attracting many young adults. There are lots of hospitality venues on the peripheries of college campuses occupied by a large number of college students as well as young adults [11].

The median ratio of indoor PM_2.5_ concentration in the post-ban to pre-ban period was 1.51 for billiards, 0.29 for pubs and 0.26 for computer game rooms. Since billiard halls were not included as a target place for the smoking ban, because they are classified as a sports facility, rather than a public place, observation of non-significant changes in PM_2.5_ concentration was not surprising.

Pubs and computer game rooms in Korea have been notorious for their potentially high SHS levels [12], making policy makers include them as major target sites for a complete smoking ban. Although the differences in PM_2.5_ concentration between pre- and post-ban period was not statistically significant, after controlling for area and the number of ventilators, our results show that, if the ban is applied, the PM_2.5_ concentrations are likely to decrease. However, in the case of computer game rooms, when the effect of enforcement of policy was further adjusted for the number of smokers, PM_2.5_ levels were increased significantly.

Our finding of the lack of reduction in PM_2.5_ concentration in computer game rooms and pubs was noteworthy. This phenomenon may be explained by an imbalance of enforcement at pubs and computer game rooms on the boundaries of college campuses, compared to the inner city [13]. A recent (2013) Web-based survey, including 5691 students (response rate 26%) and 2051 faculty/staff (response rate 43%) in the USA. reported that there was limited exposure to smoke near campus building entrances while exposure near campus boundaries was observed by most students (77%) and faculty/staff (55%) [14]. This implies that the smoking ban on campus can lead to smoking activity shifting to the campus periphery, which was similar to the balloon effect observed in our study.

Tobacco-free policies on college campuses are an emerging trend globally. For example, between September 2013 and May 2014, 1309 college students at eight public 4-year institutions across California in the USA, which has a range of policies regarding smoking (smoke-free indoors only, designated outdoor smoking areas, smoke-free, and tobacco-free), reported that stronger policies were associated with fewer students reporting exposure to secondhand smoke or seeing someone smoke on campus. It was also reported that comprehensive tobacco-free policies were effective in reducing exposure to smoking and intention to smoke on campus [15]. A recent Japanese study also demonstrated that the ban on smoking in campus served as a motivator for smokers to reduce smoking [16].

In this study, we evaluated the impact of a smoking ban policy in enclosed hospitality venues located at the periphery of college campuses. Our results revealed no significant change in PM_2.5_ levels between pre-ban and post-ban in billiard halls and there was a likely decrease of indoor PM_2.5_ in pubs and computer game rooms where the law has been implemented in campus towns, but such changes were not statistically significant yet. Compared to previous studies conducted in Spain that reported a considerable reduction in PM_2.5_ concentration levels after implementing a smoking ban on medical campuses [17], the impact of smoke-free legislation over the period has been insufficient at the peripheries of college campuses.

In short, we still could observe PM_2.5_ concentrations in pubs and computer game rooms, which should not contain SHS under the current Korean smoking ban legislation. Thus, our study indicates that compliance with the legislation must be improved more for pubs and computer game rooms although the impact of smoking ban policy was highly positive. Our study also implies that billiard venues should be included as a nonsmoking place considering their high SHS levels. Smoke-free legislation, when completely enforced, is highly effective in improving air quality and reducing the levels of indoor PM_2.5_ concentration levels [18]. Strict enforcement and periodic monitoring play a key role in the successful implementation of smoking bans.

The strength of this study was the simultaneous evaluation of the impact of a smoke-free policy on secondhand smoke exposure in different types of venues on campus peripheries in several campus towns. However, our study has several limitations. Although we used a standardized operation protocol for our one time point monitoring of PM_2.5_, there might be some differences in PM_2.5_ levels for the same point at different moments, *i.e.*, time of day, or day of the week, even though we conducted monitoring after 4 pm only during weekdays. However, we considered such differences were random. Also the small sample size limits generalization of our findings. A future study with expended sample size with a higher follow-up rate may be necessary to evaluate spatial variations of the impact of smoking ban policy at venues on the peripheries of all college campuses in Korea.

## 5. Conclusions

Indoor PM_2.5_ concentration levels on the peripheries of college campuses likely decreased 1 year after implementation of the law banning smoking, however, the differences were not significant yet. Thus, our study provides evidence for the need to introduce more rigorous policy initiatives aimed at encouraging a complete smoking ban, especially in billiard halls as well as pubs, and computer game rooms located on campus peripheries.

## References

[1] WHO, Global Health Observatory (GHO) Data. http://www.who.int/gho/phe/secondhand_smoke/en/.

[2] Ministry of Health & Welfare Law for the Promotion of Nation’s Health Enforcement Ordinance Article 6. http://www.law.go.kr/lsInfoP.do?lsiSeq=149119#AJAX.

[3] WHO Framework Convention on Tobacco Control Guidelines on Protection from Exposure to Tobacco Smoke. http://www.who.int/fctc/cop/art%208%20guidelines_english.pdf?ua=1.

[4] Statistics Korea The Census on Establishments. http://kosis.kr/ups/ups_01List.jsp.

[5] Kimberly W., Kari H., Sandra H., Niaman N., Alex W. (2006). Characteristics of social smoking among college students. J. Am. Coll. Health.

[6] Berg C.J., An L.C., Thomas J.L., Lust K.A., Sanem J.R., Swan D.W., Ahluwalia J.S. (2011). Smoking patterns, attitudes and motives: Unique characteristics among 2-year *versus* 4-year college students. Health Educ. Res..

[7] Kim S.R. (2012). Smoking prevalence and the association between smoking and sociodemographic factors using the Korea National Health and Nutrition Examination Survey data 2008 to 2010. Tob. Use Insights.

[8] Son H.K., Jung U.Y., Park K.S., Kam S., Park S.K., Lee W.K. (2009). The factors implicated when an individual starts to smoke again after a 6 month cessation. J. Prev. Med. Public Health.

[9] Business Information Analysis Corporation RJR Young Adult Motivational Research. https://industrydocuments.library.ucsf.edu/tobacco/docs/#id=lxwj0045.

[10] Sepe E., Ling P.M., Glantz S.A. (2002). Smooth moves: Bar and nightclub tobacco promotions that target young adults. Am. J. Public Health.

[11] Official Korea Tourism Organization Enjoying the Hongik University (Hongdae) Club Day. http://asiaenglish.visitkorea.or.kr/ena/SI/SI_EN_3_6.jsp?cid=309372.

[12] Kim S.R., Sohn J.Y., Lee K.Y. (2010). Exposure to particulate matters (PM_2.5_) and airborne nicotine in computer game rooms after implementation of smoke-free legislation in South Korea. Nicotine Tob. Res..

[13] Jin Y., Wang L., Lu B., Ferketich A.K. (2014). Secondhand smoke exposure, indoor smoking bans and smoking-related knowledge in China. Int. J. Environ. Res. Public Health.

[14] Braverman M.T., Hoogesteger L.A., Johnson J.A. (2015). Predictors of support among students, faculty and staff for a smoke-free university campus. Prev. Med..

[15] Fallin A., Roditis M., Glantz S.A. (2015). Association of campus tobacco policies with secondhand smoke exposure, intention to smoke on campus, and attitudes about outdoor smoking restrictions. Am. J. Public Health.

[16] Ohmi H., Okizaki T., Meadows M., Terayama K., Mochizuki Y. (2013). An exploratory analysis of the impact of a university campus smoking ban on staff and student smoking habits in Japan. Tob. Induc. Dis..

[17] Xisca S., Montse B., Cristina M., Marcela F., Esther C., Esteve S., Jose M.M., Esteve F. (2014). Impact of tobacco control policies in hospitals: Evaluation of a national smoke-free campus ban in Spain. Prev. Med. Rep..

[18] Azagba S., Kennedy R.D., Baskerville N.B. (2015). Smoke-free school policy and exposure to second-hand smoke: A quasi-experimental analysis. Nicotine Tob. Res..

